# The Doctor in the Law Courts
*An address to the Bristol Medico-Chirurgical Society, 10th April 1963.


**Published:** 1963-07

**Authors:** 


### THE DOCTOR IN THE LAW COURTS*

BY

LORD JUSTICE DONOVAN

When, many months ago, I agreed to the suggestion of my friend Dr. MacLachlan, that I should come and address you, and in turn proposed the subject of the address, I thought in my ignorance that this particular field was fairly virgin country. Recent perusal of the publications of the Medico-Legal Society have revealed to me that this is not so. Doctors have explored it and so have counsel. But I was comforted by finding that no Judge—so far as I can ascertain—has dealt with the matter as I propose to deal with it tonight; and since the ultimate end of all evidence is to persuade the person who hears it into accepting it, the reaction of the Judge, to this kind of evidence or that, to this way of presenting it or the other, and to the manner in which it is given, may have its own interest. There is no test of the quality either of evidence or of advocacy comparable to being on the receiving end of it.

I begin by a word about the distinction between our two professions. The patient comes to you as a sufferer. Your sympathy as well as your skill is enlisted on his behalf and your one desire is to help him. We too get innocent sufferers in the Law Courts, and if we possibly can we help them. Unfortunately we get other kinds of persons as well. We get the man who has committed serious crime; we get the man who has committed fraud. We get the man who is manufacturing a case for damages against his employers. And we get the man who thinks he can get damages out of his doctor for negligence although the doctor may have exercised all reasonable skill and care on his behalf. You no doubt have black sheep among your patients too; but you don't have to punish them, or publicly expose them. Nor do you stand between a man and his fellow subject, be he employer or doctor, from whom the man is seeking to extract a large sum for damages. We have to be constantly on the alert to see that crime does not pay, so far as that can ever be achieved, and that false claims do not succeed. The result is that we in the law have on some occasions to be hard, and always to be on our guard against being deceived. This in turn leads us to scrutinise evidence very carefully, to reject assertions which are not satisfactorily proved, and to reject the defence in many criminal cases "I didn't mean to do it". In this way it is very easy for the law to get the reputation of being rather harsh, hidebound, irresponsive to new ideas, and behind the times generally. This has its impact upon the relations between our two professions. For we in our turn have at times to listen to medical evidence which I believe would surprise you even more than it surprises us; and on those occasions we may say things that earn us the reputation of being impatient of medical opinion if we don't happen to like it.

The truth is, however, that Judges value honest medical opinion; that we could not do our job properly without it; and that it is only in a minority of cases that medical evidence gives rise to that acerbity of comment from the Bench which makes good news.

Some of you will have had experience already of giving evidence in a Court of Law. Some of you will not. All of you may. All of you no doubt will wish to keep the experience down to a minimum. It interferes with your time table, sometimes very grievously; opposing counsel are tiresome and sometimes, you may think, objectionable, and the Judge sometimes openly and pointedly critical. None of this would I deny. It springs from the very nature of our work which I have tried to describe.

* An address to the Bristol Medico-Chirurgical Society, 10th April 1963.

A healthy but not purblind scepticism is one of the instruments we have to use in our daily probe for truth. It connotes no real hostility, particularly not to the doctor witness.

Is there any advice which the lawyer can tender to the doctor to make the doctor's sojourn in the Courts less irksome—perhaps (who knows) even pleasurable as a break in the medical round? Without giving a categorical answer to that question I think I can, out of experience on the Bench, make some suggestions in the hope that they may help.

Take the Civil Courts first. The doctor never knows when he may be called there to give evidence. It may be on behalf of a patient who has met with an accident or contracted an illness for which he is blaming somebody else. It may be as a consultant called to give specialist evidence on diagnosis or prognosis, or evidence concerning the alleged negligence of a fellow member of the profession. It may be, alas, as the defendant himself to an action for negligence.

I am sure that most of you keep full and accurate case records of your patients' attendances upon you or yours upon them. If any of you do not, then do please do so as from tomorrow. If it should happen to you that you find yourself in Court in one of the capacities I have mentioned, it will be of the greatest help to the Court to see and read these full contemporaneous records. By that I do not mean that every time Mrs. Jones comes to your surgery you must write a treatise about her complaint. Apart from any other consideration that would be impossible in a busy practice. What it does mean is that you should note her call, her complaint, and the treatment given. You may say "Well *of course* we should do that". But I must regretfully tell you that time after time doctors appear in the Law Courts and have to confess "Yes, I did see her more often than appears on the card, but I omitted to make any entry". You may depend upon it that Counsel on the opposing side do not fail to make capital, if they can, out of the omission; and if the doctor is the defendant himself, being accused of negligence, a medical card with such omissions in it does not help him.

The evidence you may be called upon to give may simply be evidence of fact, or it may be expert evidence consisting of your medical opinion. Whichever kind of evidence you give, the observance of certain simple rules will help all round. (1) Answer only the question which is put to you, and resist any temptation to over-elaborate. (2) While you cannot wholly avoid technical medical terms, give the simple lay equivalent at the same time. Thus if you speak of a periorbital haematoma say that it means a black eye. (3) Remember that your duty is to the Court and not to the side that has called you as a witness, and be perfectly impartial and objective. The result of the case should mean nothing to you. Of course if you should be the defendant in an action for negligence the result may mean a great deal to you; but even then if it can be seen that you are completely objective and completely frank, it will do you a lot of good. (4) In cross-examination you are bound to be asked a question to which a simple answer of Yes or No will, to your knowledge, be misleading. It is then very natural to hesitate, and appear unwilling to give a simple answer to what on the face of it is a simple question. In such a case say at once and quite boldly something like this "The literal answer is Yes—or No—but the literal answer will be misleading to the Court unless I qualify it. May I do so?" No counsel is going to say "No" to that question, and no Court would let the matter rest there if he did. Or again you may be asked a question the simple answer to which may imply some default on your part which would be unjustified. Take the question "Knowing that the plaintiff had fallen down and hurt his leg, did you have an X-ray taken?". The simple answer may be "No". And the implication if the matter is left there is that you are at fault. Yet there may be quite good reasons for not having an X-ray examination. If so, return the answer "No", and add "May I explain why?". Your own counsel should get you to give the explanation when he re-examines you, but sometimes they do forget; and sometimes it is as well that the Court should have the explanation at once. Then, once you have given your evidence, and the solicitor on your side doesn't want to keep you to listen to the other side's doctor, ask the solicitor if you may be released. He will then get counsel to ask the Judge's permission, which is never refused if it can possibly be helped.

There is another matter upon which I ought to touch in this context. A doctor examining a plaintiff on behalf of defendants who are resisting his claim for damages for personal injuries will normally take down in writing the history of the matter from the plaintiff, and will ask him about his complaints past and present. No one expects this record to be meticulously accurate or comprehensive, yet the presence or absence of something in it may be seized upon by defending counsel in an endeavour to catch the plaintiff out. For example, a plaintiff may come to Court saying that since the accident he has suffered from severe and persistent headaches. On looking at the doctor's notes of what the plaintiff said at the time of his examination, counsel for the defendants may see that all the doctor has written down is "Headaches". Now says counsel "You didn't tell the doctor, did you, that your headaches were severe and persistent". "Yes I did", says the plaintiff. So when the doctor is called he is examined and cross-examined about this; and thus he becomes a witness of fact for one side rather than an expert. I have always thought this a little hard on the doctor who is thus unexpectedly made a partisan on a question of fact. And more often than not, all he can say is "Well, had the plaintiff used the words 'severe and persistent' I think I should have written them down", which may well prejudice the plaintiff if in fact the words were used and accidentally omitted. When I have heard doctors and plaintiffs examined and cross-examined in this kind of matter, I have often thought it would be a good plan for the doctor to read back his notes of the history of the case and the present complaints to the patient, and ask whether he wants to make any alterations or additions. It would not take long, and it might save trouble in any subsequent litigation. Again from time to time cases occur where a plaintiff claiming damages for personal injuries will try deliberately to deceive his own doctors and the defendant's doctors and the Court, into believing that he is worse than he is. I distinguish such malingering from hysteria where the patient is really only deceiving himself. Fortunately malingerers are few, but there are some, and they may confront the doctor who is called to give evidence in Court with a difficult tactical problem. He may be convinced that the plaintiff is malingering; yet if he says so, and cannot substantiate his opinion by some concrete evidence the possible result is to make the doctor appear unfair, prejudiced, and unreliable.

If a doctor suspects malingering something will have happened during his examinations of the plaintiff to make him suspect it. If there is any test you can make at the time which will reveal whether the patient is deliberately trying to deceive, you should make the test, and if the result is positive then as soon as the examination is over make careful notes against the day when proof may be demanded. If however the matter never gets beyond suspicion it would be better not to volunteer that suspicion in evidence. But your answers may suggest to Counsel that malingering is suspected. If so the question may asked of you, "You are not suggesting, doctor, are you, that the plaintiff is malingering?" If you really do suspect it, it is best, I think, to say "I *do* suspect it, but I am not certain". No Counsel can leave the matter there, and the next question must come "Why do you suspect it?" Then you must give your reasons. Of course play may be made afterwards that you have been over-suspicious and so on. Nevertheless you will have done your duty to the Court by being completely candid, and one never knows, something else may come out in the course of the trial to show that you were right.

The civil Courts include those dealing with Divorce and Probate. Under Rules of Court, doctors called Medical Inspectors are appointed in certain cases to examine suitors and report to the Court. These are cases where one spouse is claiming a decree of nullity on the ground of sexual incapacity, or a decree of divorce on the ground of the other spouse's wilful refusal to consummate the marriage. The doctor so appointed examines the petitioning spouse and sometimes the other one as well, and makes a written report to the Court. Sometimes he may be called to give oral evidence in support of his opinion. Beyond mentioning this procedure, I have no special observations to make upon it.

A doctor may also be called as witness in the Divorce Court when one spouse is seeking a divorce on the ground of the other's cruelty. The doctor may have been the family doctor and the petitioning spouse may call him to prove treatment for bruises and so on, and to say that while the spouses were together the petitioner's health deteriorated, and as soon as they separated it at once improved. If the petition is defended you may depend on it that you will be cross-examined at length. But you can't go far wrong if you observe the rules I have suggested.

The Probate Division of the High Court deals among other things with contested wills. From time to time some of you may be in attendance upon an elderly patient whose soundness of mind may be suspect. You will probably be treating him or her, however, for purely bodily as opposed to mental ills. Nevertheless you may afterwards find yourselves in the witness box being asked to say whether in your opinion he or she was sufficiently sound in mind to make a last will. Before this happens the solicitors for the executors, as well as the solicitors for the disappointed relatives may have got in touch with you, may have asked your opinion, and may have indicated that you will be wanted to give evidence for one side or the other in accordance with your opinion.

You are not, I might remind you, obliged to give any opinion or any proof of evidence in advance. You may take up the standpoint if you wish that if either side subpoena you, you will attend and give the Court your opinion. But if you *are* of a definite opinion either way, I see no reason why you should not say so at this earlier stage. But in the witness box you are likely to be closely cross-examined as to the foundation for it. Hence the extreme importance of contemporaneous notes. If you have any reason to suspect that an elderly patient is making a will (for example you may be asked to witness it) and that his or her mental capacity may hereafter be called in question, then again *do* take notes at the time. If I tell you what testamentary capacity consists of you will gather what points you have to watch. To be valid, a will must be made by a testator who is, at the time of making it, of sound disposing mind, memory and understanding. He must be able to comprehend and recollect the extent of his property and the nature of the claims others have upon his bounty, particularly if he is going to disregard some of them. It is essential that no disorder of the mind shall poison his affections, or pervert his sense of right, or prevent the exercise of his natural faculties, and that no insane delusion shall influence the disposition of his property.

Eccentricity alone is not incapacity. Generally speaking the law presumes sanity; but if the will is challenged in this respect then those who propound the will must prove the testator's sanity. That is subject to this, that where habitual insanity does not exist, the proof of actual insanity at the time of making the will must come from those who assert it. A good will can be made during a lucid interval, and a lucid interval does not mean that a testator must be restored altogether to his pristine soundness of intellect.

From this summary, you will see that what the doctor should look for on occasions like these are the presence or absence of any defect or reason; the presence or absence of delusions; senile decay; complete or partial loss of memory. And he should remember that although a person's mental power may have become substandard generally, he may still retain sufficient intelligence to understand the testamentary act in its different bearings. Indeed it is surprising how quick and comprehending some old people can become when it is a question of disposing of their property or money.

A doctor has no privilege to refuse to answer relevant questions put to him in the witness box, save the two privileges we all have, namely that we are not obliged to incriminate ourselves, and that we may refuse to disclose communications between ourselves and our legal advisers relating to the suit in hand. But a doctor may not, I fear, refuse to disclose in a Court of law confidential communications made to him by a patient, nor refuse to disclose the nature of the patient's illness. In 1920 there was a case in which a wife sued her husband for divorce on the ground of the communication to her by him of venereal disease. The husband's doctor was subpoenaed and when he got into the witness box he pointed out to the Judge that under Statutory regulations regulating the then National Scheme for dealing with venereal disease absolute secrecy was imposed upon the doctor. The Court held however that that did not apply to the giving of evidence, since the higher duty of doing justice over-rode it. The doctor then gave evidence that he had treated the husband for syphilis.

This, however, does not mean that any interrogator in Court, be he Counsel or solicitor or litigant in person, has a right to ask fishing questions of a doctor concerning his patient and demand an answer. The question must be relevant, and even if it is relevant the Judge has an overriding discretion to disallow it if he thinks it will serve no useful purpose, or if he thinks more harm than good is likely to result from demanding an answer. It is this ultimate discretion in the Judge which perhaps has kept this troublesome question within reasonable bounds so far.

I now desire to speak briefly of artificial insemination and the problems that it raises for doctors. So far as I know it has not yet landed any doctor in Court, but one day it is almost sure to. A doctor performing this operation is under the same obligation to use reasonable skill and care as with any other operation; and if he fails to do so, and damage to the patient results, he will be liable to an action for negligence.

Furthermore if the question ever has to be decided, there is little doubt that children born as the result of insemination by what is known as A.I.D. as opposed to A.I.H. would be regarded as illegitimate. But in the very nature of things the child so begotten is treated as legitimate and one of the family. What then as regards family estates or funds which are settled and which devolve only upon "issue" which means legitimate issue?

If a doctor is concerned in the production of illegitimate issue by A.I.D., and those illegitimate issue take property or funds which ought, in default of lawful issue, to descend to collaterals, what is the doctor's position? He certainly could be made the target of an action for conspiracy at the suit of these collaterals if they discovered the true position. No doubt it is unlikely that they ever would. And the risk could be materially reduced, though not entirely eliminated, if the doctor took from the two spouses concerned a written assurance that no property or funds would be affected by the birth of a child to the wife by A.I.D.

But over and above this, what advice is tendered by a doctor in these cases as to the registration of the birth? If the husband is registered as the father, that is perjury; and if the doctor advises such a false registration he is at once involved in criminal liability under the Perjury Act. Yet the wife can hardly register some unknown donor of the semen as the father, if she describes him as "unknown" the whole design of the appearance of legitimacy is defeated. What the solution of this difficulty is I do not know. But I think if you are asked about the registration of the birth of a child born or to be born as the result of A.I.D. you would be wise to reply that this raises legal issues rather than medical ones and is therefore primarily for the lawyer.

Now I come to the case of a doctor who is the defendant in an action for negligence at the suit of a patient. He will have to give evidence himself as a rule, and what I have already said about giving evidence applies. Claims against doctors for damages for alleged negligence have increased since the coming of the National Health Service and the Legal Aid scheme. And there may have been times when you have thought the dice were somewhat loaded against you; and that the successful treatment of patients was going to be made very difficult if you had always to have in mind the prospect of a lawsuit.

The law is that a doctor must bring to his task a reasonable degree of skill and knowledge and exercise a reasonable degree of care. What is a reasonable degree of care must of course depend upon the facts of each particular case. If the normally accepted practice prevailing at the time is followed the doctor is not liable for damages because he failed to employ some esoteric piece of knowledge of recent discovery even though an article about it may have appeared in the *Lancet*. As my colleague Lord Denning has put it, "It would be putting too high a burden on medical men to say they must read every article in the medical press". In another case he used these words: "We should be doing a disservice to the community if we imposed on hospitals and doctors liability for everything that happened to go wrong. Doctors would tend to think more of their own safety than of the good of their patients, initiative would be stifled and confidence shaken. A proper sense of proportion requires us to have regard to the conditions in which doctors and hospitals work. We must insist on due care for the patient, but we must not condemn as negligence that which is only misadventure".

I feel sure that all my other colleagues in the Court of Appeal would subscribe to those sentiments and act accordingly. Of course if it should happen on occasion that the right operation is performed but on the wrong patient there is nothing much that we can do.

I now leave the civil and turn to the criminal Courts. The guidance I have tried to give as regards evidence in a civil Court applies equally to the criminal Court. But the nature of the evidence a doctor is frequently asked to give in a criminal Court calls for special mention.

Our law presumes that every man is sane, and intends the natural consequences of his acts. If I aim a loaded revolver at a man's head and pull the trigger the natural consequence is that I will kill him, and if I do the law presumes that I meant to. But this presumption is rebuttable. My defence may be that I am insane. The law admits such a defence, for under the law insane persons are not punished for what they do. But the law casts the burden of proof upon me, and since 1843 it has stated with some particularity what it is I have to prove.

In the year 1843 one Daniel McNaghten, the son of a Glasgow carpenter, came to London. For eight years he had been suffering from the delusion that he was constantly being followed by Government spies. Apart from this delusion he did not appear insane; he had his own woodworking business in Scotland, worked hard, and was honest with his customers. On 20th January 1843 Mr. Edward Drummond who was secretary to Sir Robert Peel left the Privy Council office in Downing Street, and went to the family Bank near Charing Cross. He left the Bank and was close to a coffee house where Charing Cross station now stands when McNaghten came up from behind, took out a pistol, and shot Mr. Drummond in the side. In hospital the ball was removed from Mr. Drummond's body, but septicaemia set in and he died five days after the shooting. Popular legend has it that McNaghten mistook Mr. Drummond for Sir Robert Peel himself, and shot him as a protest against the Corn Laws. For this suggestion there is no evidence. The strong probability is that McNaghten took Mr. Drummond for one of the horde of Government spies that he imagined were constantly around him.

McNaghten's trial for murder took place at the Old Bailey in March 1843. He was tried before a jury by three Judges, Lord Chief Justice Tindal, Mr. Justice Williams, and Mr. Justice Coleridge. The defence was insanity and numerous doctors were called, including the doctor of Newgate prison. They all said McNaghten was insane. The Solicitor-General who was prosecuting said he was not in a position to call any rebutting evidence, and would therefore no longer press for a verdict of guilty.

The Lord Chief Justice directed the jury as follows:

"The question to be decided is whether at the time the act was committed the prisoner had or had not the use of his understanding, so as to know he was doing a wrong or a wicked act. If you are of opinion that the prisoner was not sensible at the time he would be entitled to a verdict in his favour; but if on the contrary you are of opinion that when he committed the act he was in a sound state of mind then your verdict must be against him".

The jury returned a verdict of Not Guilty on the ground of insanity. To-day of course he would be Guilty but insane.

The verdict gave rise to public dissatisfaction, because the expectation had been that the trial would disclose seditious activities of the Anti-Corn Law League, and some were disappointed that it did nothing of the sort. But it gave rise to debates in the House of Lords as to what extent insanity should be a defence to a criminal charge, and the Lord Chancellor decided to settle the matter by putting a series of questions to the Judges. They were five in all. It is only one answer which I need recall tonight. It was this:

"We have to submit our opinion that jurors ought to be told in all cases that every man is to be presumed sane and to possess a sufficient degree of reason to be responsible for his crimes, until the contrary is proved to their satisfaction; and that to establish a defence on the ground of insanity it must be clearly proved that the time of committing the act, the accused was labouring under such a defect of reason, due to disease of the mind as not to know the nature and quality of the act he was doing; or if he did know it that he did not know that what he was doing was wrong".

This has been the law ever since, and thus did the carpenter's son from Glasgow achieve legal immortality.

Over 100 years later, in 1957, the Government of the day, disturbed by the prospect that a bill to abolish capital punishment might be passed by the combined votes of the Labour opposition and their supporters on the Government Benches, brought in their own bill which resulted in the Homicide Act of that year. Under this only a few murders were retained as capital offences (murders in the course of theft, murders by explosion and shooting, murders in resisting arrest, murders of a police officer or prison officer in the course of their duties). The Government also by the same Act introduced the defence of diminished responsibility in a case of murder. That crime can be reduced to manslaughter if the accused proves that at the time he was suffering from such abnormality of mind (whether arising from a condition of arrested or retarded development of mind or any inherent causes or induced by disease or injury), as substantially impaired his mental responsibility for his acts and omissions in doing or being a party to the killing. The McNaghten rules and this new defence of diminished responsibility account for a great deal of the medical evidence called in criminal Courts today, and much of that evidence is given by psychiatrists.

Before 1957 when all murders were capital a psychiatrist called to give evidence for the defence in support of a plea of insanity sometimes had an unhappy time. Unless the alleged insanity was pretty obvious the psychiatrist had an uphill battle. The murder may have been revolting and atrocious, and Judge and jury, to say nothing of prosecuting Counsel, were all assembled as representing the community to see that due retribution was exacted if justice so required. And since in the class of case I am considering the deed was admitted, the onus was on the prisoner to prove that he should escape retribution because he was insane at the time he did the killing. The hope of establishing this defence rested mainly on the psychiatrist and the evidence he would give.

Now the language of psychiatry is not the language of the ordinary man such as finds his way into the jury box, or even onto the Bench. And the ordinary man before 1957 and even today has considerable difficulty in understanding afflictions which do not manifest themselves in bodily symptoms, particularly when they are given strange and, to him, incomprehensible names. He knew that insanity yields to no satisfactory definition and unless the psychiatrist's evidence could be expressed in clear and simple language and could be illuminated by examples or illustrations which struck an answering chord in the minds of the jury, then their reaction—particularly under the direction of a sceptical Judge—was apt to be unfavourable. They would say to themselves "We are really being asked to find this man insane because no sane man could commit so horrible a crime". In other words it is simply reasoning backwards from the deed.

On the other hand if the psychiatrist's evidence was clear, fair, expressed so far as possible in simple language, and avoided the appearance of dogmatism, the jury would be more impressed and more inclined to stretch a point. They might then not bring the full rigour of the McNaghten test to bear, but might simply ask themselves as a matter of common sense "Is this man mad or not?"

The position has I think changed since the Homicide Act of 1957. Now most murders are non-capital and the strain of trying a man for his life has to that extent departed. Furthermore the new defence of diminished responsibility is bringing more doctors and more psychiatrists into the criminal Courts. And while judges and juries still do not have the vocabulary of psychiatry at their full command, nevertheless with increasing experience we continue to learn. And the value of psychiatric evidence is more and more appreciated.

But it must still be good evidence; and to be good evidence it must be well prepared. The doctor giving it must know the case history. He will be allowed to refresh his memory from his contemporaneous notes of his examinations of the accused, but it is always well to refresh one's memory before actually going into the witness box. It creates a bad impression and devalues your evidence if you obviously have not refreshed your mind about the case before giving evidence. You should specify if you can the data upon which you have arrived at your conclusion. Under hostile cross-examination keep cool and try to avoid dogmatism if you can. The question may be asked "Is it not possible that you are mistaken, etc. etc." and the question may be coupled with some extract from a text book which appears to contradict you. If you are absolutely confident that you are right there is no reason why you should not say so and refuse to budge.

But if, as I suppose must sometimes happen in this field, the opposite view is just barely possible it is better to say so, and add "but it is highly improbable"—if that be your view. The greater the impression of impartiality and fairness you give the greater weight will Judge and jury attach to your evidence.

Beware too of the question "*May* this be so?" Many a doctor, knowing in his heart that it is *not* so will refuse at first to concede even the bare possibility. Then when at last he is forced to do so the impression may, quite wrongly, be given that he is partisan. Such a question if asked is better answered by some such reply as this: "The possibility may exist, but if it does it is remote. The overwhelming probability is the other way."

It is a good plan once the psychiatrist has written the report on which his medical evidence will be based to turn himself into a Devil's advocate, and try to assail it as he would if he were cross-examining counsel. This can hardly fail to be good preparation for the cross-examination when it comes. To summarise therefore: know your facts, be objective, explain your technical terms in lay language as far as you can, be ready to meet technical criticism, avoid dogmatism unless you have to be dogmatic. This may sound like a policy of perfection but the nearer the psychiatrist can get to it, the greater his value as a witness, and the greater his authority in the witness box.

Now I know the medical profession in general dislikes the McNaghten rules and considers they are wholly out of date and were probably out of date when they were first promulgated 120 years ago. For it is said, "There are people who know what they are doing, and who know it is wrong, but through mental affliction simply cannot control their will and cannot control their impulse to do that very thing. These people fall outside the McNaghten rules and they are accordingly punished for doing something they just cannot help doing. Yet by any just assessment these people are as insane as those who get the protection of the rules". Well, this controversy is of long standing, and it is outside the scope of my address to renew it. Those who are interested will find both sides of the question elaborately and fully debated in the Report of the Royal Commission on Capital Punishment published in 1953.

I desire however to draw your attention to this development. In 1960 a man named Byrne raped a girl in a hostel and then beheaded her. At his trial for murder the medical evidence showed that from an early age he had been subject to perverted sexual desires and that he found it very difficult and perhaps impossible to resist putting them into practice on occasions; and that the killing had been done under such an impulse on this occasion. Putting these sexual addictions on one side, the accused was normal in every other respect. His defence was diminished responsibility. The trial Judge held that that defence was not open to him as a matter of law. He directed the jury that if Parliament had intended that the irresistible impulses of a sex maniac were a good defence, and reduced murder to manslaughter, Parliament would have said so in express terms. The jury accordingly convicted Byrne of murder.

The Court of Criminal Appeal held that the Judge's direction was wrong. They said that what had to be decided by the jury first of all was whether, in the words of the Act, there was abnormality of mind present in Byrne. That meant, *inter alia*, having to consider his ability to exercise his will-power to control his physical acts in accordance with a rational judgment. Byrne admittedly could not do that, and accordingly he suffered from an abnormality of mind and such an abnormality as substantially diminished his responsibility.

In essence therefore this was a defence based on uncontrollable impulse. So long as that amounts to an abnormality of mind substantially diminishing one's responsibility, this new defence is open. It eventually succeeded in Byrne's case, the Court of Criminal Appeal reducing the verdict to one of manslaughter.

But diminished responsibility is a defence only in the case of murder; and even then it does no more than reduce the crime to manslaughter. It may, but often does not, reduce the punishment. You may ask, if uncontrollable impulse is a defence why is it not a complete defence, leading, if it be established, to a verdict of acquittal; and why is the defence not available in the case of all crimes? What is the justification for punishing a man who through mental abnormality cannot help doing what he did?

The answer is, I fear, the purely practical one that the law cannot afford always to be logical, and that society in the present state of crime, and with its overriding responsibility for the protection of the citizen, cannot admit such a defence generally for fear of its abuse. What test is to reveal to us in satisfactory measure which impulse was irresistible and which merely not resisted? Let me illustrate the point by reference to a case of capital murder which was the last such case I ever tried at the Old Bailey.

A man named Smith with a previous conviction had stolen some scaffold clips from a building site, and put them in a bag in the boot of his car. His car was stopped by a police constable who knew the man, and who asked him had he anything in the boot and if so what? Smith made some evasive reply, and the officer told him to draw over to the kerb. Smith proceeded to do so with the constable walking beside the car and holding on to it. Smith then started to accelerate. The constable flung himself across the bonnet shouting to Smith to stop. Instead he went faster up the road. He swerved towards three oncoming cars in succession, though he had no need to, and bumped the officer against each one in turn. Then he bumped him against a fourth car and the officer fell off. He was dead, with horrible injuries, when he was picked up.

In his defence Smith said "I never intended to do that officer any harm. I merely wanted to get away and get rid of the stolen property". Now if the defence of uncontrollable impulse were generally admitted by our law, I feel sure that it would have have been put forward in that case, and perhaps successfully. You can almost hear it being said "I don't know what came over me. I was suddenly seized with an impulse to get away and to get rid of the officer. I simply had to obey this impulse. I just couldn't resist it. I am very sorry". What could the prosecution do if such a defence were allowed? At the end of the day it is always for the prosecution to prove guilt. The Judge would be bound to say to the jury, "If you think it may reasonably be true that there was this uncontrollable impulse, then you should acquit". Yet if Smith had been acquitted and gone free after killing the constable in the way I have described, the public conscience would surely have been deeply shocked.

Bear with us therefore if we in the law appear to you at times to be unenlightened, and perhaps even reactionary. We recognise, of course, that if all crime were the result of some illness in the individual which medical treatment could cure, that would be ideal. Away would go the prisons and the criminal courts, and their place would be taken by hospitals. But alas, we are not yet in sight of the promised land. Hatred and despair, jealousy and lust, envy and cruelty, these human failings yield as yet to no known specific; and they are the mainsprings of crime. Crime goes on increasing in this country, and it pays, for well under one half of the indictable crimes committed are followed by the conviction of their authors. In this state of affairs society can scarcely be blamed if it declines to make defences available for those who are caught which, by reducing the chances of conviction, will make crime more attractive still, and the lot of the honest citizen more sorry. Nevertheless, the country has to its credit gone a long way recently to meet the case of those who are mentally sick rather than wicked; for the Mental Health Act of 1959 now enables a Judge instead of punishing an offender to send him to hospital for medical treatment. There are reformers, even among lawyers, and to them some credit for this is due; but I feel sure the bulk of the credit is due to members of your own profession.

